# A Provident Scheme

**Published:** 1894-06-23

**Authors:** 


### THE INSTITUTIONAL WORKSHOP.

### HOSPITAL FINANCE.

### VIII.—A PROVIDENT SCHEME.

There is little room for doubt that sick assurance wisely organised on a sufficiently comprehensive basis would command the almost universal support of the regular wage earning and small salaried members of the community. We believe that the true strength of the hospitals lies in this class. But to claim their support on charitable grounds is awkward in theory, and has not proved very successful in practice. The contributors are probably deeply indebted to the institutions for benefits received or expected, in which case the contributions are ridiculously inadequate; while, on the other hand, a considerable proportion of those from whom contributions are asked are straining every nerve to keep clear of free medical assistance themselves, and have neither power nor inclination to aid in providing it for their less thrifty neighbours.

To gain general confidence, and prove a solid support in time of illness, sick assurance must provide for three distinct classes of patients—in-patients, who can only be treated satisfactorily in a hospital; home patients, who cannot or do not wish to be moved; and out-patients, who are able to attend either at the hospital or at the doctor's house. The third class includes also what may be termed consulting patients, who need the advice of a specialist.

Co-operation among the various branches of the hospital, medical, and nursing services could alone enable these three classes to be dealt with by one insurance, and as it is obviously impossible for any man to forecast the nature of the assistance he will need, this co-operation must be reckoned indispensable.

Selecting London for the field of operations, it will be well to sketch very briefly in what way a general insurance of the lower middle classes, aided by the hearty co-operation of the medical profession, would work for good to both.

Upon the inevitable central council of the proposed association, representing the interests of all concerned, would devolve the work of organizing sub-centres on a like representative basis in each district, and of regulating alike the subscription of members and the fees paid for their attendance and treatment. The adjustment of receipts and payments in a proportion which should satisfy just claims and exclude any risk of deficit, would be a matter of some difficulty, and must be subject, even under the most cautious calculation, to occasional revision. That the association should be entirely self-supporting, and that the rate of fees should be uniform for the metropolis, are the two most important points in this connection.

In each district a paid secretary would register members, receive their subscriptions, and attend at a central office to furnish information and send immediate aid where necessary. The first step in the organisation of each district would be the enlistment of such of the medical practitioners living within its limits as would consent to act as associate doctors. Absolute freedom would be allowed to any duly qualified practitioner to inscribe his name on the list, the object of the association being not to interfere in the smallest degree with medical practice, but to render the fee easy of payment by the patient, and a matter of certainty to the doctor. It is needless to say that the fees guaranteed should be sufficient to invite men of good standing, if not of large practice to offer their services. To young men of zeal and ability, on quitting a hospital appointment, such an opening would be eagerly welcomed, and there are many older men who would not despise an introduction to their poorer neighbours were they freed from the painful harass of charging ill-spared and grudgingly-paid fees. Liberty of choice in the selection of their doctor is valued even more by the poor than by the rich, and medical assurance will never get a firm hold on the people until a much wider scope is allowed to individual preference than is customary under any existing system. A list of associate doctors with their addresses and hours of consultation would be kept open to the inspection of members at the central office in each district, and an order from the secretary would be the only formality necessary in making application to the one selected. The associate doctor would not only visit patients in their homes, but receive at his own house those who were able to attend, and he would be asked to fix a convenient hour in the evening for the reception of patients who are at work during the day.

In addition to those medical practitioners who undertake the work of visiting and receiving patients, the association would procure the co-operation of certain leading physicians and surgeons to act as consultants when occasion should require. No part of the free hospital and dispensary machinery is so popular among the poor as the facilities which they afford for obtaining a "good opinion." Many of the most flagrant acts of imposition in the acceptance of medical relief are committed in the struggle to obtain this expensive luxury by highly respectable people who have never in their lives been the possessors of a clear two guineas to spend on themselves. It can hardly be doubted that many eminent specialists would aid in the task of unpauperising London by devoting a small part of their time at a reduced fee to cases selected and sent on to them by the associate doctors. The strain upon the special departments of the hospitals would be relieved in proportion as the system gained ground, and the just annoyance caused to doctors and hospital authorities by the pertinacity of the well-to-do out-patient would gradually disappear. The advantage of obtaining the consultation without the fatigue and humiliation of waiting for hours among a crowd of suffering applicants would be readily recognized by patients, and a way of restoration to health would be opened to many who are now completely debarred from good advice.

It is not, however, only the specialist who would be asked to co-operate with the association. The leading general practitioners would no doubt consent to aid the associate doctors in the event of any grave complication arising with home patients. It is the ability to fall back on an older head, or it may be simply to compare experiences, which give so great an advantage to the dispensary surgeon or parish doctor's assistant in critical cases. It is common enough among the very poor to hear of two or even three doctors consulting over the sick bed. Among the lower middle-class this very rarely, if ever, occurs. The expense entailed by a severe illness, with the prospect of a heavy doctor's bill at the end, debars the patient from suggesting it, and his doctor is too sensible to worry him by proposing an impossibility. Yet inter-communication between the successful doctor and his brother practitioners could be only to the advantage of both, not to mention the patient. The busy physician might dread, it is true, the additional labour of even an occasional consultation, but before long he would find his labours lightened as one after another of his gratuitous patients joined the association. The hard-working governess, the under-paid curate, the struggling musician and artist, whose fees he now declines, would be placed beyond the need of his generosity by payments scarcely felt in the season of health. The fee paid by the association for a consultation would be less than that offered by rich patients, but it would be such as fairly to remunerate him for his time, and the widening of his clinical experience by cases of exceptional interest, would be valued by most men enthusiastic in their profession.

Home patients of small means would in this way be enabled to count upon medical attention fully equal in value to that which would be theirs without question had they no means at all.

But there are many cases which can only be dealt with satisfactorily in hospitals. A system of hearty co-operation with hospital authorities would enable such cases to be transported in an ambulance without difficulty or delay to the nearest hospital suited to the needs of the patient.

A certificate from the associate doctor in charge of the case, counter-signed by the district secretary, would take the place of a hospital letter, and if the hospital applied to had no vacant bed, there would be no delay in procuring admission for an urgent case elsewhere. Both special and general hospitals are very unequally distributed over London, and it would be found impracticable to confine each district to those hospitals lying within its limits.

For each patient thus admitted a fixed sum per diem would be paid by the association. The amount of this sum, in common with the amount of the fees paid to doctors, would be fixed by the Central Committee, representing the hospital authorities, doctors, and members. It has been estimated that "On an average £70 per occupied bed should suffice to supply the best administered hospital with every hospital requirement, a liberal dietary, and an efficient staff."* About four shillings a day would then represent the fair proportion of each in-patient's expenses calculated on the total ordinary expenditure. This amount paid on any considerable number of patients would doubtless entail a larger subscription than would readily be forthcoming, and a reasonable proportion of the full expense, say three-fourths, might be accepted as a sufficient contribution. Such an amount paid by even a quarter of the present in-patients in London hospitals would suffice to ease all financial difficulties.

To place the paying patients in a special ward and exempt them, should they desire it, from the assiduities of the medical student, need not entail either additional trouble or expense.

It may be objected that some who now never dream of availing themselves of hospital treatment might be induced under the new system to offer themselves as patients. It has been already urged that the struggling middle class who will not be pauperised and cannot pay for proper advice or nursing have fully as much claim on our consideration as the most picturesque beggar in the streets. A member of a large family in a small house, or lying alone hopelessly neglected in a squalid lodging, how many so situated die simply for want of the commonest care?

To rescue these brave workers from their present anomalous and unfair position, to raise the regular wage-earner from a degrading dependence on public charity, and to protect the great medical charities for the really destitute, who will never be wanting in our midst, are aims for which it were well worth while to lay aside class interests and professional jealousy. By co-operation alone can they be achieved.

* "Hospitals of the World," p. 168. (H. C. Burdett.)

